# Flavonoid Glycosides and Hydroxycinnamic Acid Derivatives in Baby Leaf Rapeseed From White and Yellow Flowering Cultivars With Repeated Harvest in a 2-Years Field Study

**DOI:** 10.3389/fpls.2019.00355

**Published:** 2019-04-02

**Authors:** Marie Groenbaek, Erik Tybirk, Susanne Neugart, Ulrik Kraemer Sundekilde, Monika Schreiner, Hanne Lakkenborg Kristensen

**Affiliations:** ^1^Department of Food Science, Aarhus University, Aarhus, Denmark; ^2^Knold & Top ApS., Odder, Denmark; ^3^Department Plant Quality and Food Security, Leibniz Institute of Vegetable and Ornamental Crops e. V., Grossbeeren, Germany

**Keywords:** *Brassica napus* L. var. *oleifera*, baby leaf, repeated harvest, flavonoid glycosides, hydroxycinnamic acids, life cycle

## Abstract

Recently, new annual and biennial cultivars of rapeseed with white flowers have been introduced to the baby leaf market. The white flower trait has been bred into modern cultivars of yellow flowering rapeseed. In baby leaf production, it is common practice to perform several cuts of the same plants, thereby harvesting regrown material. Seven white and yellow flowering annual and biennial rapeseed cultivars were harvested as baby leaves, baby leaf re-growths, and intact plants in order to investigate the content of flavonoid glycosides and hydroxycinnamic acid derivatives. The field experiment was conducted over two consecutive years to obtain seasonal differences. The yields and levels of flavonoid glycosides and hydroxycinnamic acids were higher in 2016 than 2017, due to higher temperatures and radiation. Within the growing stage, the effects of flower color, cultivar, and life cycle on flavonoid glycosides and hydroxycinnamic acids varied; however, in general, life cycle was the main influence that resulted in elevated levels of flavonoid glycosides and hydroxycinnamic acids in biennial cultivars, compared to annual cultivars. The effects of the growing stage differed between years, and were influenced by climatic conditions. In conclusion, the choice of life cycle (annual or biennial cultivars) and seasonal effects was of major influence, overruling the effect of developmental stage on the content of flavonoid glycosides and hydroxycinnamic acids.

## Introduction

Rapeseed (*Brassica napus* L. var. *oleifera*) is known and used worldwide for the production of oil. In northern Europe, the annual (spring) cultivars are normally sown in the spring and harvested in the summer. The biennial (winter) cultivars are sown in late summer and harvested in summer the following year. Therefore, different cultivars have diverse qualities that are linked to their life cycle, such as day-length or vernalization requirements for flowering and winter hardiness. Around the world, different varieties of *B. napus* are also utilized as leafy products for human consumption, but this is not common practice in Western countries (Farag et al., 2013; Lee et al., 2013). Recently, new annual and biennial cultivars of rapeseed with white flowers have been introduced to the baby leaf market in the US and southern Europe. The white flower trait has been bred into modern cultivars of yellow flowering rapeseed, originally with the aim of reducing the need for insecticides as pollen beetles (*Meligethes aeneus*), cabbage seedpod weevils (*Ceutorhynchus assimilis*), and brassica pod midges (*Dasineura brassicae)* are less attracted to white than to yellow colored flowers. It is not yet clear if the differences in flower color are linked to the differences in the flavonol glycosides and hydroxycinnamic acid derivatives in rapeseed.

Differences between cultivars of *Brassica oleracea* have been studied previously, and it was determined that genetic diversity can cause substantial variation in the content of polyphenols such as flavonoid glycosides and hydroxycinnamic acid derivatives (Mageney et al., 2017; Neugart et al., 2018). However, no study has yet investigated the variation in the flavonoid glycoside and hydroxycinnamic acid derivative content between rapeseed cultivars. The focus so far has been on the identification of flavonols in the plant material, with a specific emphasis on the seed content of hydroxycinnamic acids such as sinapic and ferulic acids (Liu et al., 2012; Farag et al., 2013; Shao et al., 2014).

Flavonoid glycosides and hydroxycinnamic acid derivatives are shielding compounds that are produced in leaves to protect against damage from high levels of solar radiation, particularly ultraviolet (UV) radiation. Previous findings indicate that season and light level have an impact on the composition and content of flavonoid glycosides. An increase of the content of quercetin and isorhamnetin aglycones was found as temperature and radiation levels decreased in several cultivars of curly kale (*B. oleracea* L. var. *sabellica*), whereas the content of kaempferol aglycones decreased during the same period (Schmidt et al., 2010a). Furthermore, studies have indicated that flavonoid glycosides contribute to the prevention of cancer, atherosclerosis, and chronic inflammation in humans by slowing down oxidative degradation (Cartea et al., 2011).

It is common practice in the production of certain baby leaf species to perform several harvests of the same plants, thereby harvesting the regrown material from already established plants in order to increase yields and obtain a sustainable production (Hall et al., 2015; Kristensen and Stavridou, 2017). However, the effects of this production method have only been investigated to a limited degree and no studies have been conducted on the effects of repeated harvests on flavonoid glycosides and hydroxycinnamic acid contents in rapeseed baby leaves, or in any other *Brassica* leafy green (Hall et al., 2015; Sasaki et al., 2015).

The objectives of the study were to investigate whether the composition and content of flavonoid glycosides and hydroxycinnamic acid derivatives is influenced by the cultivar (five white flowering and two yellow flowering rapeseed cultivars), including qualities ascribed to the life cycle of the plants (annual/biennial). We aimed to test the effects of the growing stage, specifically the baby leaves (Stage I), re-grown baby leaves (Stage II_cut_), and intact plants (Stage II_uncut_), on the composition and content of flavonoid glycosides and hydroxycinnamic acid derivatives in a field experiment. In order to address the influence of seasonal variation and rate of plant development, the study was conducted with shifted sowing and harvesting dates over two consecutive years.

## Materials and Methods

### Plant Material

Two cultivars of yellow flowering rapeseed (“Fenja” from NPZ-Lembke, Holtsee, Germany and “Labrador” from KWS SAAT AG, Einbeck, Germany) and five cultivars of white flowering rapeseed (“Lysidé,” “SilverShadow,” “Jadak,” “Witt,” and “Lilput” from Knold & Top ApS., Odder, Denmark) were tested in an organically farmed experimental field. The field is situated at the Department of Food Science in Aarslev, Denmark (55°18′N, 10°27′E) on a sandy loam soil (Typic Agrudalf), which contained 118 and 141 kg mineral nitrogen (N) ha^−1^ and 1.1 and 0.7 kg sulfur (S) ha^−1^ in the top 25 cm soil layer in 2016 and 2017, respectively. One month before sowing, 80 kg N and 0.9 kg S ha^−1^ in 2016 and 40 kg N and 0.2 kg S ha^−1^ in 2017 were dispersed as dried chicken manure (4-1-3, DLG, Odense, Denmark). In addition, 6 kg S ha^−1^ was applied in 2017 as kieserite (K+S Kali GmbH, Germany). Plots of 1.6 m × 5 m were organized in a randomized complete block design with three replicates, and rapeseed was sown in 10 rows to have 800 plants per square meter, which is common practice (Kristensen and Stavridou, 2017). Sowing dates were July 20th, 2016 and August 28th, 2017. The outermost extra plots were created to eliminate border effects, and an insect net (0.8 mm mesh size) covered the plants during the entire experiment. Data on global radiation and on maximum and minimum temperatures were provided by a Danish Meteorological Institute weather station situated in the experimental field. The growing degree days (GDD) were calculated as (T_max_ + T_min_)/2–T_base_ per day, where T_base_ was set as 0.9 °C for both biennial and annual cultivars (Vigil et al., 1997). The accumulated GDD is the sum of daily GDD from sowing until harvest.

Prior to the harvest, the developmental stage of the baby leaf rapeseed (Stage I) was assessed using the BBCH-scale (Lancashire et al., 1991). The Stage I harvest took place 27 and 29 days after sowing (DAS) in 2016 and 2017, respectively. One square meter per plot was harvested just above the apical meristem using a hedge trimmer with a tray attached. Yields were assessed by the total weight of the harvested biomass, and the plants were stored for two days in slightly perforated plastic bags at 0.5°C and 98% relative humidity prior to preparation for chemical analysis. The intact plants (Stage II_uncut_) were evaluated using the BBCH-scale prior to harvest of another square meter per plot at 40 DAS (2016) and 60 DAS (2017). At the same time, a repeated harvest was undertaken on the regrown leaves (Stage II_cut_) from the square meters harvested at 27 and 29 DAS. The plants were stored as mentioned above. Yields from these harvests were not assessed. Results for glucosinolate content and composition, along with a sensory evaluation of the rapeseed cultivars, can be found in Groenbaek et al. (submitted).

### Chemical Analyses

Approximately 150 g of fully unfolded leaves of different sizes with 2–3 cm stalk were frozen in liquid N and freeze-dried in a CHRIST Gamma 1–20 freeze-dryer (Martin Christ Gefriertrocknungsanlagen GmbH, Osterode am Harz, Germany), from which dry matter (DM) was calculated. Freeze-dried plant material was ground to 0.5 mm using a Retsch Mixer Mill MM 200 (SkanLab ApS., Slangerup, Denmark) and stored in a freezer at −24°C prior to further analysis.

To analyze the flavonol glycosides and hydroxycinnamic acid derivatives, the samples were extracted as described in Groenbaek et al. (2014), with the following modifications. In analytical duplicates, 20 mg of freeze-dried, ground powder was extracted with 600 μL of 60% aqueous methanol for 1 h with vigorous agitation at room temperature. Samples were centrifuged at 19,000 g for 10 min and the supernatant collected in a new tube. The extraction procedure was repeated with 400 and 200 μL of 60% aqueous methanol for 20 and 10 min, respectively. The supernatants were combined and evaporated to dryness with rotation. The residue was dissolved in 200 μL of distilled water. The extract was then filtered through Corning® Costar® Spin-X® plastic centrifuge tube filters with a cellulose acetate membrane (Sigma Aldrich Chemical Co., St. Louis, MO, USA) for high-performance liquid chromatography (HPLC) analysis.

The HPLC-diode array detector-electrospray ionization-mass spectrometry (HPLC-DAD-ESI-MS^n^) method for the tentative identification and quantification of the flavonol glycosides and hydroxycinnamic acid derivatives in the different cultivars of rapeseed was undertaken using a Series 1100 HPLC (Agilent, Waldbronn, Germany) with a degasser, binary pump, auto sampler, column oven, and photodiode array detector. An ion trap (Agilent series 1100 MSD) with an ESI ion source was used in negative ionization mode as the mass spectrometer. The parameters for the HPLC-DAD-ESI-MS^n^ were as described by Neugart et al. (2012). Mass fragments used for the identification of the compounds were as previously published (Schmidt et al., 2010b). The standards caffeoylquinic acid (Chlorogenic acid), quercetin-3-*O*-glucoside (for quercetin glycosides), and kaempferol-3-*O*-glucoside (for kaempferol glycosides) were used to obtain an external calibration curve ranging from 0.1 to 10 mg 100 mL^−1^ for a semi-quantitative approach.

### Statistical Analysis

All univariate statistical calculations were performed using SAS (SAS Institute Inc., Cary, NC, release 9.4, 2000) using a general linear model to test least squares means separations (*p* ≤ 0.05) of flavonoid glycoside and hydroxycinnamic acid derivative content due to flower color, cultivars, life cycle (annual/biennial), year and life cycle × year. When comparing effects from cultivar, growing stage, and cultivar × growing stage on flavonoid glycoside and hydroxycinnamic acid derivative content the mixed model procedure was used to test the least squares means separations (*p* ≤ 0.05), as harvest was undertaken on the same plants and in the same plot twice. Growing stage was the repeated variable. When needed, data were transformed in order to achieve a normal distribution and homogeneity of variance. Principal Component Analysis (PCA) was performed using PLSToolbox (Eigenvector Research, Inc., Manson, WA, release 8.6.2) to identify differences in the flavonoid glycosides and hydroxycinnamic acid derivatives (*n* = 22) found in the seven cultivars. The data were autoscaled prior to PCA modeling.

## Results

### Yields and Growth

The fresh weight yield of baby leaf rapeseed (Stage I) differed between cultivars (within year), life cycle, and year (Table 1). Biennial cultivars had a higher yield than the annuals, and the 2016 harvest resulted in approximately twice as high yields than the 2017 harvest. A lower number of plants per square meter was observed in 2016. The BBCH scale evaluation at Stage II_uncut_ was influenced greatly by the cultivar in 2016, as the annual cultivars had undergone formation of buds. Climatic data from the growing periods are shown in Supplementary Figure S1.

**Table 1:** Yield in fresh weight (FW; Mg ha^−1^), dry matter percentage (DM%), number of plants per m^2^, and BBCH-scale code of seven white or yellow flowering annual or biennial rapeseed cultivars harvested as baby leaves (Stage I), 27 and 29 days after sowing and as intact plants (Stage II_uncut_), 40 and 60 days after sowing in 2016 and 2017, respectively.

**Year**	**Life cycle**	**Cultivar (cv)**	**Yield (Mg FW ha^**−1**^) stage I**	**DM% stage I**	**#plants m^**−2**^ stage I**	**BBCH-scale stage I**	**BBCH-scale stage II_**uncut**_**
2016	Annual	Lysidé (w)*^*a*^*	8.1 ± 1.7	10.8 ± 0.5	242 ± 79	13.4 ± 0.1	28.6 ± 14.9
		Silvershadow (w)	15.1 ± 2.4	10.3 ± 0.1	399 ± 166	13.3 ± 0.3	56.7 ± 1.2
		Fenja (y)	13.6 ± 1.7	10.2 ± 0.3	403 ± 46	13.1 ± 0.1	56.8 ± 2.3
	Biennial	Jadak (w)	16.0 ± 2.1	10.4 ± 0.5	350 ± 59	13.7 ± 0.6	16.0 ± 0.0
		Witt (w)	14.8 ± 0.7	10.4 ± 0.4	472 ± 97	13.5 ± 0.5	15.7 ± 0.3
		Lilput (w)	13.6 ± 3.8	10.2 ± 0.7	359 ± 84	13.6 ± 0.6	16.0 ± 0.0
		Labrador (y)	16.5 ± 1.3	10.6 ± 0.4	446 ± 58	13.2 ± 0.4	15.7 ± 0.4
*Cv effects*			^**^^b^	^*^	*NS*	*NS*	^**^
2017	Annual	Lysidé (w)					16.0 ± 0.0
		Silvershadow (w)	4.7 ± 0.7	10.1 ± 0.0	535 ± 56	13.1 ± 0.1	15.9 ± 0.1
		Fenja (y)	3.8 ± 0.6	11.2 ± 0.4	663 ± 3	13.3 ± 0.1	15.9 ± 0.1
	Biennial	Jadak (w)	8.5 ± 0.7	10.7 ± 0.1	748 ± 84	14.0 ± 0.5	15.3 ± 1.0
		Witt (w)	7.3 ± 010	10.8 ± 0.2	689 ± 17	13.8 ± 0.0	15.9 ± 0.1
		Lilput (w)	5.9 ± 0.2	10.9 ± 0.0	510 ± 51	13.8 ± 0.3	15.4 ± 0.8
		Labrador (y)	7.8 ± 1.3	10.3 ± 0.1	662 ± 15	14.0 ± 0.0	15.7 ± 0.1
*Cv effects*			^**^	^***^	^***^	^*^	*NS*
**SIGNIFICANCE**							
Cv			NS	NS	NS	NS (*p* = 0.0567)	^*^
Life cycle			^***^	NS	NS	^**^	^***^
Year			^***^	NS	^***^	^*^	^**^
Life cycle * year			NS	NS	NS	^**^	^***^

*Values of yield, DM% and #plants m^-2^ are means (n = 3) ± standard deviation, values of BBCH are means of 4 ± standard deviation. Cultivar (cv) significance obtained from f(x) = cv + block both within year and between years; Life cycle and year significance obtained from f(x) = life cycle + year life + cycle × year block*.

a *w, white flower color; y, yellow flower color*.

b* p ≤ 0.05;

** p < 0.01;

*** *p < 0.001; NS, not significant. BBCH-scale code were analyzed with Kruskal-Wallis test*.

### Effect of Cultivar and Flower Color on the Flavonoid Glycoside and Hydroxycinnamic Acid Derivative Content

We detected 13 flavonoid glycosides and eight main hydroxycinnamic acid derivatives plus one unknown (Tables 2–4), in accordance with previous findings for the leaves and seeds of *B. napus* L. (Farag et al., 2013; Shao et al., 2014). Acylated and non-acylated kaempferol triglycosides were the most abundant, followed by the hydroxycinnamic acid derivative and quercetin glycoside group. The acylation of flavonoid glycosides with hydroxycinnamic acids (*p*-coumaric, caffeic, ferulic, hydroxyferulic, and sinapic acid) is a well-known phenomenon in *Brassica* species. For the first time, kaempferol-3-*O*-hydroxyferuoyl-sophoroside-7-*O*-D-glucosides and several hydroxycinnamic acids have been identified in *B. napus* (genome AACC), though they have previously been described in *B. oleracea* (genome CC) (Neugart et al., 2014) and *B. rapa* (genome AA) (Heinze et al., 2018). The only isorhamnetin glycoside found was isorhamnetin-3-*O*-D-glucoside-7-*O*-D-glucoside, which was found in trace quantities and therefore the data are not presented.

**Table 2:** Content of kaempferol glycosides (mg g^−1^ DM) of seven white or yellow flowering, annual or biennial rapeseed cultivars harvested as baby leaves (Stage I) 27 and 29 days after sowing in 2016 and 2017, respectively.

**Year**	**Life cycle**	**Cultivar (cv)**	**k-3*-O-*soph-7*-O-*glc*^**a**^***	**k-3*-O-*cou-soph-7*-O-*D-glc**	**k-3*-O-*caf-soph-7*-O-*D-glc**	**k-3*-O-*fer-soph-7*-O-*D-glc**	**k-3*-O-*hfer-soph-7*-O-*D-glc**	**k-3*-O-*sin-soph-7*-O-*D-glc**	**k-3*-O-*sin-soph-7*-O-*diglc**	**Total kaempferol glycosides**
2016	Annual	Lysidé (w)*^*b*^*	0.74 ± 0.18	0.20 ± 0.01	1.14 ± 0.19	0.90 ± 0.14	1.17 ± 0.25	1.12 ± 0.18	0.47 ± 0.06	5.74 ± 1.01
		Silvershadow (w)	0.89 ± 0.19	0.07 ± 0.01	0.78 ± 0.08	0.87 ± 0.07	1.46 ± 0.17	0.61 ± 0.05	0.37 ± 0.05	5.05 ± 0.59
		Fenja (y)	0.55 ± 0.20	0.27 ± 0.09	1.20 ± 0.46	1.14 ± 0.42	2.02 ± 0.86	1.39 ± 0.53	0.28 ± 0.12	6.86 ± 2.59
	Biennial	Jadak (w)	0.37 ± 0.02	0.41 ± 0.07	1.33 ± 0.20	0.96 ± 0.12	1.62 ± 0.18	1.18 ± 0.12	0.17 ± 0.02	6.04 ± 0.70
		Witt (w)	0.37 ± 0.08	0.17 ± 0.03	1.03 ± 0.07	0.97 ± 0.08	1.59 ± 0.10	0.92 ± 0.07	0.18 ± 0.02	5.23 ± 0.41
		Lilput (w)	0.26 ± 0.05	0.29 ± 0.08	1.54 ± 0.34	1.11 ± 0.19	1.55 ± 0.40	1.29 ± 0.19	0.17 ± 0.04	6.21 ± 1.27
		Labrador (y)	0.32 ± 0.01	0.45 ± 0.09	1.69 ± 0.20	1.22 ± 0.12	2.20 ± 0.18	1.35 ± 0.13	0.24 ± 0.08	7.48 ± 0.79
*Flower color effect*			*NS^*c*^*	*NS*	*NS*	^*^	^**^	^*^	*NS*	^*^
2017	Annual	Lysidé (w)								
		Silvershadow (w)	0.33 ± 0.05	0.26 ± 0.02	0.66 ± 0.04	0.93 ± 0.05	1.43 ± 0.09	0.79 ± 0.05	0.25 ± 0.02	4.65 ± 0.27
		Fenja (y)	0.10 ± 0.00	0.14 ± 0.07	0.63 ± 0.02	0.67 ± 0.03	0.93 ± 0.05	1.11 ± 0.10	0.14 ± 0.01	3.70 ± 0.14
	Biennial	Jadak (w)	0.07 ± 0.00	0.15 ± 0.01	0.72 ± 0.04	0.64 ± 0.02	0.84 ± 0.09	1.07 ± 0.06	0.16 ± 0.01	3.64 ± 0.22
		Witt (w)	0.05 ± 0.01	0.10 ± 0.05	0.48 ± 0.03	0.60 ± 0.05	0.77 ± 0.05	0.82 ± 0.07	0.13 ± 0.01	2.95 ± 0.17
		Lilput (w)	0.07 ± 0.01	0.11 ± 0.01	0.91 ± 0.03	0.80 ± 0.02	0.87 ± 0.06	1.19 ± 0.04	0.12 ± 0.02	4.07 ± 0.18
		Labrador (y)	0.03 ± 0.00	0.14 ± 0.00	0.72 ± 0.04	0.63 ± 0.02	0.81 ± 0.06	0.91 ± 0.03	0.18 ± 0.01	3.43 ± 0.15
*Flower color effect*			*NS*	*NS*	*NS*	*NS*	*NS*	*NS*	*NS*	*NS*
**SIGNIFICANCE**										
Cv			*^*d*^*	NS	NS	NS	NS	^***^	^***^	NS
Life cycle			*^*d*^*	NS	^*^	NS	NS	NS	^***^	NS
Year			*^*d*^*	^*^	^***^	^***^	^**^	NS	^***^	^***^
Life cycle ^*^ year			*^*d*^*	^**^	NS	NS	NS	NS	NS	NS

*Values are means (n = 3) ± standard deviation. Cv significance obtained from f(x) = cv + block; Flower color effect obtained from f(x) = flower color + block within each year; Life cycle and year significance obtained from f(x) = life cycle + year + life cycle × year block*.

a *K, kaempferol; soph, sophoroside; cou, coumaroyl; glc, glucoside; caf, caffeoyl; fer, feruloyl; hfer, hydroxyferuloyl; sin, sinapoyl; diglc, diglycoside. Values of k-3-O-caf-soph-7-O-D-glc and k-3-O-hfer-soph-7-O-D-glc are sums of two isomers*.

b *w, white flower color; y, yellow flower color*.

c* p ≤ 0.05;

** p < 0.01;

*** *p < 0.001; NS, not significant*.

*^d^Data could not obtain normal distribution or homogeneity of variance*.

**Table 3:** Content of hydroxycinnamic acids (mg g^−1^ DM) of seven white or yellow flowering, annual or biennial rapeseed cultivars harvested as baby leaves (Stage I) 27 and 29 days after sowing in 2016 and 2017, respectively.

**Year**	**Life cycle**	**Cultivar (cv)**	**Ferulic acid**	**Sinapic acid**	**Caffeoyl- quinic acid**	**Caffeoyl glucoside**	**Disinapoyl-gentiobiose**	**Sinapoyl-feruloyl- gentiobiose**	**Trisinapoyl-gentiobiose**	**Disinapoyl-feruloyl-gentiobiose**	**Total hydroxy-cinnamic acids**
2016	Annual	Lysidé (w)*^*a*^*	0.79 ± 0.09	1.20 ± 0.07	0.46 ± 0.05	0.13 ± 0.02	0.06 ± 0.05	0.03 ± 0.03	0.02 ± 0.01	0.02 ± 0.00	2.79 ± 0.26
		Silvershadow (w)	0.77 ± 0.08	0.90 ± 0.07	0.15 ± 0.02	0.18 ± 0.03	0.03 ± 0.01	0.01 ± 0.00	0.01 ± 0.00	0.01 ± 0.00	2.11 ± 0.20
		Fenja (y)	0.58 ± 0.24	0.94 ± 0.36	0.32 ± 0.12	0.12 ± 0.05	0.22 ± 0.08	0.01 ± 0.01	0.04 ± 0.02	0.04 ± 0.02	2.47 ± 0.95
	Biennial	Jadak (w)	0.26 ± 0.08	0.60 ± 0.15	0.46 ± 0.06	0.35 ± 0.04	0.40 ± 0.04	0.00 ± 0.00	0.03 ± 0.00	0.03 ± 0.00	2.43 ± 0.36
		Witt (w)	0.52 ± 0.05	0.75 ± 0.04	0.27 ± 0.03	0.25 ± 0.03	0.29 ± 0.05	0.01 ± 0.01	0.04 ± 0.01	0.05 ± 0.01	2.37 ± 0.12
		Lilput (w)	0.27 ± 0.09	0.40 ± 0.36	0.38 ± 0.07	0.20 ± 0.04	0.55 ± 0.10	0.00 ± 0.00	0.04 ± 0.01	0.04 ± 0.01	2.27 ± 0.59
		Labrador (y)	0.41 ± 0.16	0.80 ± 0.30	0.38 ± 0.04	0.29 ± 0.03	0.51 ± 0.02	0.00 ± 0.00	0.04 ± 0.01	0.04 ± 0.00	2.80 ± 0.60
*Flower color effect*			*NS^*b*^*	*NS*	*NS*	*NS*	*NS*	*^*c*^*	*NS*	*NS*	*NS*
2017	Annual	Lysidé (w)									
		Silvershadow (w)	0.73 ± 0.06	0.95 ± 0.07	0.11 ± 0.01	0.11 ± 0.01	0.05 ± 0.00	0.00 ± 0.00	0.01 ± 0.00	0.01 ± 0.00	2.04 ± 0.15
		Fenja (y)	0.27 ± 0.01	0.45 ± 0.36	0.16 ± 0.01	0.06 ± 0.00	0.19 ± 0.01	0.01 ± 0.01	0.04 ± 0.00	0.04 ± 0.00	1.38 ± 0.31
	Biennial	Jadak (w)	0.11 ± 0.01	0.38 ± 0.01	0.30 ± 0.02	0.24 ± 0.01	0.47 ± 0.04	0.00 ± 0.00	0.03 ± 0.00	0.03 ± 0.00	1.93 ± 0.08
		Witt (w)	0.20 ± 0.02	0.47 ± 0.04	0.14 ± 0.01	0.14 ± 0.01	0.28 ± 0.03	0.00 ± 0.00	0.04 ± 0.00	0.05 ± 0.00	1.53 ± 0.13
		Lilput (w)	0.12 ± 0.01	0.27 ± 0.03	0.24 ± 0.01	0.12 ± 0.00	0.60 ± 0.04	0.00 ± 0.00	0.03 ± 0.00	0.03 ± 0.00	1.79 ± 0.11
		Labrador (y)	0.14 ± 0.01	0.42 ± 0.01	0.17 ± 0.00	0.15 ± 0.00	0.46 ± 0.03	0.00 ± 0.00	0.03 ± 0.00	0.03 ± 0.00	1.70 ± 0.06
*Flower color effect*			*NS*	*NS*	*NS*	*NS*	*NS*	*NS*	*NS*	*NS*	*NS*
**SIGNIFICANCE**											
Cv			^***^	^***^	^***^	^***^	^***^	NS	^***^	^***^	NS
Life cycle			^***^	^***^	^**^	^***^	^***^	NS	NS	NS	NS
Year			^***^	^***^	^***^	^***^	NS	NS	NS	NS	^***^
Life cycle ^*^ year			NS	NS	NS	NS	NS	NS	NS	NS	NS

*Values are means (n = 3) ± standard deviation. Cv significance obtained from f(x) = cv + block; Flower color effect obtained from f(x) = flower color + block within each year; Life cycle and year significance obtained from f(x) = life cycle + year + life cycle × year block*.

a *w, white flower color; y, yellow flower color*.

b* p ≤ 0.05;

** p < 0.01;

*** *p < 0.001; NS, not significant*.

c *Data could not obtain normal distribution or homogeneity of variance*.

**Table 4:** Content of quercetin glycosides (mg g^−1^ DM) of seven white or yellow flowering, annual or biennial rapeseed cultivars harvested as baby leaves (Stage I) 27 and 29 days after sowing in 2016 and 2017, respectively.

**Year**	**Life cycle**	**Cultivar (cv)**	**q-3*-O-*soph-7*-O-*glc*^***a***^***	**q-3*-O-*caf-soph-7*-O-*D-glc**	**q-3*-O-*sin-soph-7*-O-*D-glc**	**Total quercetin glycosides**
2016	Annual	Lysidé (w)*^*b*^*	0.04 ± 0.01	0.20 ± 0.05	0.15 ± 0.04	0.39 ± 0.09
		Silvershadow (w)	0.05 ± 0.01	0.22 ± 0.02	0.12 ± 0.02	0.39 ± 0.04
		Fenja (y)	0.06 ± 0.02	0.29 ± 0.11	0.30 ± 0.12	0.65 ± 0.25
	Biennial	Jadak (w)	0.03 ± 0.00	0.12 ± 0.03	0.11 ± 0.01	0.26 ± 0.04
		Witt (w)	0.03 ± 0.01	0.31 ± 0.04	0.20 ± 0.02	0.54 ± 0.06
		Lilput (w)	0.03 ± 0.01	0.13 ± 0.03	0.08 ± 0.02	0.24 ± 0.05
		Labrador (y)	0.04 ± 0.00	0.21 ± 0.03	0.14 ± 0.02	0.39 ± 0.05
*Flower color effect*			*NS^*c*^*	*NS*	*NS*	*NS*
2017	Annual	Lysidé (w)				
		Silvershadow (w)	0.03 ± 0.01	0.14 ± 0.01	0.21 ± 0.02	0.38 ± 0.03
		Fenja (y)	0.03 ± 0.00	0.16 ± 0.00	0.30 ± 0.02	0.49 ± 0.02
	Biennial	Jadak (w)	0.02 ± 0.00	0.07 ± 0.00	0.10 ± 0.00	0.19 ± 0.01
		Witt (w)	0.01 ± 0.00	0.14 ± 0.01	0.19 ± 0.02	0.34 ± 0.03
		Lilput (w)	0.02 ± 0.00	0.10 ± 0.00	0.09 ± 0.01	0.21 ± 0.01
		Labrador (y)	0.02 ± 0.00	0.10 ± 0.01	0.10 ± 0.01	0.22 ± 0.02
*Flower color effect*			*NS*	*NS*	*^*d*^*	*NS*
**SIGNIFICANCE**						
Cv			NS	^**^	^***^	^***^
Life cycle			^***^	^**^	^***^	^***^
Year			^***^	^***^	NS	NS
Life cycle ^*^ year			NS	NS	NS	NS

*Values are means (n = 3) ± standard deviation. Cv significance obtained from f(x) = cv + block; Flower color effect obtained from f(x) = flower color + block within each year; Life cycle and year significance obtained from f(x) = life cycle + year + life cycle × year block*.

a *Q, quercetin; soph, sophoroside; glc, glucoside; caf, caffeoyl; sin, sinapoyl*.

b *w, white flower color; y, yellow flower color*.

c* p ≤ 0.05;

** p < 0.01;

*** *p < 0.001; NS, not significant*.

d *Data could not obtain normal distribution or homogeneity of variance*.

At **Stage I** harvest in 2016, a higher level of total kaempferol glycosides were found in the yellow flowering cultivars, compared to the white flowering cultivars (Table 2). Cultivar differences for the kaempferol glycosides were limited to kaempferol-3-O-sinapoyl-sophoroside-7-*O*-diglucoside and kaempferol-3-*O*-sinapoyl-sophoroside-7-*O*-D-glucoside. There were higher levels of several of the mono-acylated kaempferol glycosides in the yellow flowering cultivars compared to the white flowering cultivars in 2016, but not in 2017 (Table 2). Cultivar effects were more pronounced within the individual hydroxycinnamic acid derivatives, with the content of ferulic, sinapic and caffeoylquinic acids; caffeoyl glucoside; disinapoylgentiobiose; trisinapoylgentiobiose; and disinapoylferuloylgentiobiose affected (Table 3). In more detail, the white flowering cultivars “Lysidé” and “Silvershadow” had the highest levels of ferulic, sinapic, and caffeoylquinic (in 2016) acids. In contrast, the highest levels of caffeoyl glucoside and disinapoylgentiobiose were found in the cultivars “Jadak” and “Lilput”. No cultivar effects on total hydroxycinnamic acids were observed, perhaps due to the divergent responses between the cultivars for the identified hydroxycinnamic acids. The yellow flowering cultivar “Fenja” had the highest content of total quercetin glycosides, mono-acylated quercetin-3-*O*-caffeoyl-sophoroside-7-*O*-D-glucoside (together with “Witt”), and quercetin-3-*O*-sinapoyl-sophoroside-7-*O*-D-glucoside (Table 4).

For the intact plants harvested at **Stage II**_**uncut**_, cultivar effects were observed for kaempferol-3-*O*-sophoroside-7-*O*-glucoside, kaempferol-3-*O*-caffeoyl-sophoroside-7-*O*-D-glucoside, kaempferol-3-*O*-sinapoyl-sophoroside-7-*O*-D-glucoside, and kaempferol-3-*O*-coumaroyl-sophoroside-7-*O*-D-glucoside (Table 5). At the same stage in 2016, higher levels of kaempferol-3-*O*-hydroxyferuloyl-sophoroside-7-*O*-D-glucoside, kaempferol-3-*O*-sinapoyl-sophoroside-7-*O*-diglucoside, and kaempferol-3-*O*-sinapoyl-sophoroside-7-*O*-D-glucoside were found in the yellow flowering cultivars. In contrast, the kaempferol-3-*O*-coumaroyl-sophoroside-7-*O*-D-glucoside content was higher in the white flowering cultivars in 2016. A flower color effect was revealed in a higher content of total hydroxycinnamic acids in the white flowering cultivars, in addition to a cultivar effect (Table 6). Cultivar effects on the individual hydroxycinnamic acids followed the same pattern that was observed at Stage I. In addition, flower color effects were observed that resulted in a higher content of ferulic and sinapic acids in the white flowering cultivars in 2016, and a higher content of trisinapoylgentiobiose in the yellow flowering cultivars in 2107. Cultivar effects were observed for total quercetin glycosides and quercetin-3-O-caffeoyl-sophoroside-7-O-D-glucoside, as well as a higher content of total quercetin glycosides and all identified quercetin glycosides, in the yellow flowering cultivars in 2016 (Table 7).

**Table 5:** Content of kaempferol glycosides (mg g^−1^ DM) of seven white or yellow flowering, annual or biennial rapeseed cultivars harvested as intact plants (Stage II_uncut_) 40 and 60 days after sowing in 2016 and 2017, respectively.

**Year**	**Life cycle**	**Cultivar (cv)**	**k-3*-O-*soph-7*-O-*glc*^***a***^***	**k-3*-O-*cou-soph-7*-O-*D-glc**	**k-3*-O-*caf-soph-7*-O-*D-glc**	**k-3*-O-*fer-soph-7*-O-*D-glc**	**k-3*-O-*hfer-soph-7*-O-*D-glc**	**k-3*-O-*sin-soph-7*-O-*D-glc**	**k-3*-O-*sin-soph-7*-O-*diglc**	**Total kaempferol glycosides**
2016	Annual	Lysidé (w)^b^	0.54 ± 0.18	0.33 ± 0.10	0.91 ± 0.21	0.89 ± 0.25	1.07 ± 0.35	0.94 ± 0.30	0.44 ± 0.11	5.11 ± 1.30
		Silvershadow (w)	0.92 ± 0.05	0.05 ± 0.01	0.66 ± 0.07	1.06 ± 0.10	1.62 ± 0.10	0.69 ± 0.03	0.29 ± 0.03	5.28 ± 0.33
		Fenja (y)	0.39 ± 0.06	0.11 ± 0.01	0.90 ± 0.11	1.03 ± 0.11	1.94 ± 0.30	1.27 ± 0.19	0.35 ± 0.07	5.98 ± 0.82
	Biennial	Jadak (w)	0.46 ± 0.08	0.16 ± 0.01	0.82 ± 0.04	1.04 ± 0.10	1.58 ± 0.11	0.83 ± 0.07	0.20 ± 0.01	5.10 ± 0.38
		Witt (w)	0.43 ± 0.02	0.48 ± 0.03	1.23 ± 0.50	0.96 ± 0.05	1.03 ± 0.79	1.19 ± 0.07	0.31 ± 0.03	5.62 ± 0.20
		Lilput (w)	0.24 ± 0.03	0.36 ± 0.06	1.08 ± 0.13	0.95 ± 0.12	1.27 ± 0.25	1.14 ± 0.15	0.31 ± 0.04	5.35 ± 0.76
		Labrador (y)	0.34 ± 0.05	0.02 ± 0.01	1.04 ± 0.08	0.31 ± 0.04	1.55 ± 0.15	1.10 ± 0.06	1.28 ± 0.24	5.65 ± 0.51
*Flower color effect*			*NS^*c*^*	^**^	*NS*	*^*d*^*	^*^	^*^	^**^	*NS*
2017	Annual	Lysidé (w)	0.37 ± 0.03	0.20 ± 0.02	0.72 ± 0.02	0.88 ± 0.01	0.88 ± 0.01	1.48 ± 0.07	0.18 ± 0.01	4.73 ± 0.05
		Silvershadow (w)	0.25 ± 0.05	0.21 ± 0.01	0.38 ± 0.01	0.76 ± 0.02	0.99 ± 0.04	0.77 ± 0.04	0.13 ± 0.02	3.49 ± 0.14
		Fenja (y)	0.15 ± 0.05	0.19 ± 0.02	0.41 ± 0.02	0.64 ± 0.02	0.89 ± 0.04	1.00 ± 0.03	0.11 ± 0.02	3.40 ± 0.12
	Biennial	Jadak (w)	0.19 ± 0.05	0.28 ± 0.04	0.79 ± 0.12	0.91 ± 0.13	1.19 ± 0.20	1.34 ± 0.20	0.19 ± 0.02	4.87 ± 0.74
		Witt (w)	0.17 ± 0.06	0.10 ± 0.09	0.61 ± 0.11	0.89 ± 0.13	1.12 ± 0.16	1.09 ± 0.08	0.15 ± 0.02	4.11 ± 0.46
		Lilput (w)	0.11 ± 0.03	0.15 ± 0.02	0.71 ± 0.07	0.79 ± 0.09	0.74 ± 0.07	1.22 ± 0.13	0.17 ± 0.01	3.89 ± 0.38
		Labrador (y)	0.14 ± 0.02	0.20 ± 0.13	0.83 ± 0.04	1.00 ± 0.04	1.33 ± 0.07	1.35 ± 0.08	0.24 ± 0.01	5.09 ± 0.04
*Flower color effect*			*NS*	*NS*	*NS*	*NS*	*NS*	*NS*	*NS*	*NS*
**SIGNIFICANCE**										
Cv			^*^	^***^	^*^	NS	NS	^***^	*^*d*^*	NS
Life cycle			^***^	NS	^***^	NS	NS	NS	NS	NS
Year			^***^	NS	^***^	NS	^***^	NS	^***^	^***^
Life cycle ^*^ year			NS	NS	NS	NS	NS	NS	NS	NS

*Values are means (n = 3) ± standard deviation. Cv significance obtained from f(x) = cv + block; Flower color effect obtained from f(x) = flower color + block within each year; Life cycle and year significance obtained from f(x) = life cycle + year + life cycle × year block*.

a *For abbreviations please consult Table 2. Values of k-3-O-caf-soph-7-O-D-glc and k-3-O-hfer-soph-7-O-D-glc are sums of two isomers*.

b *w, white flower color; y, yellow flower color*.

c NS, not significant;

* p ≤ 0.05;

** p < 0.01;

*** *p < 0.001*.

d *Data could not obtain homogeneity of variance*.

**Table 6:** Content of hydroxycinnamic acids (mg g^−1^ DM) of seven white or yellow flowering, annual or biennial rapeseed cultivars harvested as intact plants (Stage II_uncut_) 40 and 60 days after sowing in 2016 and 2017, respectively.

**Year**	**Life cycle**	**Cultivar (cv)**	**Ferulic acid**	**Sinapic acid**	**Caffeoyl- quinic acid**	**Caffeoyl glucoside**	**Disinapoyl-gentiobiose**	**Sinapoyl-feruloyl- gentiobiose**	**Trisinapoyl-gentiobiose**	**Disinapoyl-feruloyl-gentiobiose**	**Total hydroxy-cinnamic acids**
2016	Annual	Lysidé (w)*^*a*^*	0.82 ± 0.22	1.34 ± 0.36	0.67 ± 0.19	0.12 ± 0.04	0.04 ± 0.01	0.01 ± 0.00	0.04 ± 0.01	0.01 ± 0.00	3.11 ± 0.85
		Silvershadow (w)	0.72 ± 0.06	0.90 ± 0.03	0.19 ± 0.00	0.15 ± 0.00	0.00 ± 0.00	0.02 ± 0.00	0.02 ± 0.00	0.01 ± 0.00	2.04 ± 0.08
		Fenja (y)	0.55 ± 0.10	1.10 ± 0.22	0.44 ± 0.06	0.10 ± 0.02	0.09 ± 0.01	0.00 ± 0.00	0.05 ± 0.01	0.02 ± 0.00	2.48 ± 0.42
	Biennial	Jadak (w)	0.51 ± 0.02	0.97 ± 0.01	0.53 ± 0.02	0.34 ± 0.03	0.13 ± 0.01	0.00 ± 0.00	0.05 ± 0.01	0.02 ± 0.00	2.73 ± 0.11
		Witt (w)	0.67 ± 0.02	0.94 ± 0.02	0.29 ± 0.02	0.23 ± 0.02	0.09 ± 0.01	0.00 ± 0.00	0.04 ± 0.00	0.02 ± 0.00	2.42 ± 0.07
		Lilput (w)	0.53 ± 0.07	0.97 ± 0.15	0.45 ± 0.10	0.19 ± 0.04	0.20 ± 0.06	0.00 ± 0.00	0.06 ± 0.02	0.02 ± 0.01	2.72 ± 0.53
		Labrador (y)	0.02 ± 0.01	0.55 ± 0.07	0.33 ± 0.02	0.19 ± 0.01	0.13 ± 0.02	0.01 ± 0.00	0.03 ± 0.01	0.01 ± 0.00	1.42 ± 0.07
*Flower color effect*			^**^^b^	^*^	*NS*	*NS*	*NS*	*NS*	*NS*	*NS*	^*^
2017	Annual	Lysidé (w)	0.60 ± 0.03	1.18 ± 0.02	0.26 ± 0.01	0.05 ± 0.00	0.06 ± 0.00	0.00 ± 0.00	0.03 ± 0.00	0.01 ± 0.00	2.34 ± 0.06
		Silvershadow (w)	0.51 ± 0.02	0.95 ± 0.10	0.10 ± 0.01	0.07 ± 0.00	0.02 ± 0.00	0.00 ± 0.00	0.03 ± 0.01	0.01 ± 0.00	1.73 ± 0.09
		Fenja (y)	0.34 ± 0.06	0.84 ± 0.08	0.17 ± 0.02	0.03 ± 0.01	0.08 ± 0.01	0.00 ± 0.00	0.05 ± 0.00	0.01 ± 0.00	1.70 ± 0.19
	Biennial	Jadak (w)	0.29 ± 0.06	0.78 ± 0.09	0.31 ± 0.02	0.17 ± 0.01	0.17 ± 0.02	0.00 ± 0.00	0.03 ± 0.00	0.01 ± 0.00	2.05 ± 0.18
		Witt (w)	0.39 ± 0.05	0.84 ± 0.08	0.17 ± 0.01	0.11 ± 0.01	0.13 ± 0.04	0.00 ± 0.00	0.04 ± 0.01	0.02 ± 0.00	1.94 ± 0.24
		Lilput (w)	0.22 ± 0.02	0.73 ± 0.07	0.22 ± 0.01	0.09 ± 0.01	0.23 ± 0.05	0.00 ± 0.00	0.04 ± 0.02	0.01 ± 0.01	1.95 ± 0.05
		Labrador (y)	0.33 ± 0.03	0.87 ± 0.01	0.21 ± 0.00	0.13 ± 0.00	0.22 ± 0.01	0.00 ± 0.00	0.04 ± 0.00	0.02 ± 0.00	2.15 ± 0.01
*Flower color effect*			*NS*	*NS*	*NS*	*NS*	*NS*	*NS*	^*^	*NS*	*NS*
**SIGNIFICANCE**											
Cv			^**^	^***^	^***^	^***^	^***^	NS	^***^	^***^	^*^
Life cycle			^***^	^***^	NS	^***^	^***^	NS	NS	^***^	NS
Year			^**^	NS	^***^	^***^	NS	^**^	NS	NS	^**^
Life cycle ^*^ year			NS	NS	NS	NS	NS	^*^	NS	NS	NS

*Values are means (n = 3) ± standard deviation. Cv significance obtained from f(x) = cv + block; Flower color effect obtained from f(x) = flower color + block within each year; Life cycle and year significance obtained from f(x) = life cycle + year + life cycle × year block*.

a *w, white flower color; y, yellow flower color*.

b* p ≤ 0.05;

** p < 0.01;

*** *p < 0.001; NS, not significant*.

**Table 7:** Content of quercetin glycosides (mg g^−1^ DM) of seven white or yellow flowering, annual or biennial rapeseed cultivars harvested as intact plants (Stage II_uncut_) 40 and 60 days after sowing in 2016 and 2017, respectively.

**Year**	**Life cycle**	**Cultivar (cv)**	**q-3*-O-*soph-7*-O-*glc*^***a***^***	**q-3*-O-*caf-soph-7*-O-*D-glc**	**q-3*-O-*sin-soph-7*-O-*D-glc**	**Total quercetin glycosides**
2016	Annual	Lysidé (w)*^*b*^*	0.03 ± 0.01	0.06 ± 0.01	0.08 ± 0.03	0.18 ± 0.05
		Silvershadow (w)	0.05 ± 0.00	0.07 ± 0.01	0.09 ± 0.00	0.21 ± 0.00
		Fenja (y)	0.05 ± 0.01	0.07 ± 0.02	0.13 ± 0.03	0.25 ± 0.05
	Biennial	Jadak (w)	0.03 ± 0.00	0.06 ± 0.01	0.09 ± 0.01	0.18 ± 0.02
		Witt (w)	0.03 ± 0.00	0.11 ± 0.01	0.12 ± 0.01	0.26 ± 0.03
		Lilput (w)	0.03 ± 0.01	0.05 ± 0.01	0.04 ± 0.01	0.11 ± 0.02
		Labrador (y)	0.05 ± 0.01	0.18 ± 0.03	0.10 ± 0.02	0.32 ± 0.01
*Flower color effect*			^**^^c^	^*^	^*^	^**^
2017	Annual	Lysidé (w)	0.02 ± 0.00	0.08 ± 0.00	0.18 ± 0.02	0.28 ± 0.02
		Silvershadow (w)	0.01 ± 0.00	0.04 ± 0.00	0.10 ± 0.01	0.15 ± 0.01
		Fenja (y)	0.02 ± 0.01	0.04 ± 0.01	0.10 ± 0.03	0.15 ± 0.04
	Biennial	Jadak (w)	0.02 ± 0.00	0.03 ± 0.01	0.06 ± 0.02	0.11 ± 0.03
		Witt (w)	0.02 ± 0.01	0.06 ± 0.01	0.12 ± 0.02	0.20 ± 0.04
		Lilput (w)	0.01 ± 0.00	0.03 ± 0.00	0.04 ± 0.00	0.08 ± 0.00
		Labrador (y)	0.02 ± 0.00	0.05 ± 0.01	0.08 ± 0.01	0.14 ± 0.02
*Flower color effect*			*NS*	*NS*	*NS*	*NS*
**SIGNIFICANCE**						
Cv			NS	^*^	*^*d*^*	^***^
Life cycle			^*^	NS	^**^	NS
Year			^***^	^***^	NS	^*^
Life cycle * year			NS	NS	NS	NS

*Values are means (n = 3) ± standard deviation. Cv significance obtained from f(x) = cv + block; Flower color effect obtained from f(x) = flower color + block within each year; Life cycle and year significance obtained from f(x) = life cycle + year + life cycle × year block*.

a *For abbreviations please consult Table 4*.

b *w, white flower color; y, yellow flower color*.

c* p ≤ 0.05;

** p < 0.01;

*** *p < 0.001; NS, not significant*.

d *Data could not obtain homogeneity of variance*.

The effects of the cultivar on total kaempferol glycosides content and on all identified kaempferol glycosides content were determined when baby leaf re-growth was harvested (**Stage II**_**cut**_) (Table 8). The yellow flowering cultivar “Labrador” in particular responded with a high content of the mono-acylated triglycosides of kaempferol, whereas the white flowering cultivars “Lysidé” and “Silvershadow” contained higher levels of the non-acylated kaempferol-3-*O*-sophoroside-7-O-glucoside and the kaempferol tetraglycoside kaempferol-3-O-sinapoyl-sophoroside-7-*O*-diglucoside. A flower color effect was only observed for kaempferol-3-*O*-coumaroyl-sophoroside-7-*O*-D-glucoside, with a higher content in white flowering cultivars in 2017, as seen at Stage II_uncut_ in 2016 (Table 5). Cultivar effects on total hydroxycinnamic acids were observed (Table 9). Moreover, the effects of cultivar on the content of individual hydroxycinnamic acids at Stage II_cut_ followed the pattern observed at Stage I and Stage II_uncut_. Effects of flower color were observed in 2016 for trisinapoylgentiobiose and disinapoylferuloylgentiobiose, with higher levels in the yellow flowering cultivars. Finally, cultivar effects were also observed for the total quercetin glycosides, quercetin-3-*O*-sophoroside-7-*O*-glucoside, and quercetin-3-*O*-sinapoyl-sophoroside-7-*O*-D-glucoside content, with all three annual cultivars, together with “Witt,” having the highest content (Table 10).

**Table 8:** Content of kaempferol glycosides (mg g^−1^ DM) of seven white or yellow flowering, annual or biennial rapeseed cultivars harvested baby leaf re-growth (Stage II_cut_) 40 and 60 days after sowing in 2016 and 2017, respectively.

**Year**	**Life cycle**	**Cultivar (cv)**	**k-3*-O-*soph-7*-O-*glc*^***a***^***	**k-3*-O-*cou-soph-7*-O-*D-glc**	**k-3*-O-*caf-soph-7*-O-*D-glc**	**k-3*-O-*fer-soph-7*-O-*D-glc**	**k-3*-O-*hfer-soph-7*-O-*D-glc**	**k-3*-O-*sin-soph-7*-O-*D-glc**	**k-3*-O-*sin-soph-7*-O-*diglc**	**Total kaempferol glycosides**
2016	Annual	Lysidé (w)*^*b*^*	0.59 ± 0.12	0.18 ± 0.18	0.93 ± 0.18	0.81 ± 0.22	1.19 ± 0.35	0.98 ± 0.25	0.55 ± 0.13	5.23 ± 1.19
		Silvershadow (w)								
		Fenja (y)	0.29 ± 0.02	0.04 ± 0.00	0.70 ± 0.01	0.65 ± 0.01	1.44 ± 0.01	0.90 ± 0.02	0.30 ± 0.01	4.32 ± 0.01
	Biennial	Jadak (w)	0.29 ± 0.02	0.24 ± 0.13	0.88 ± 0.07	0.83 ± 0.04	1.77 ± 0.10	1.04 ± 0.04	0.29 ± 0.01	5.34 ± 0.33
		Witt (w)	0.37 ± 0.02	0.06 ± 0.01	0.72 ± 0.03	0.91 ± 0.05	1.70 ± 0.06	0.70 ± 0.06	0.21 ± 0.02	4.67 ± 0.16
		Lilput (w)	0.20 ± 0.01	0.21 ± 0.12	1.04 ± 0.06	0.89 ± 0.02	1.65 ± 0.15	1.09 ± 0.05	0.29 ± 0.01	5.36 ± 0.37
		Labrador (y)	0.29 ± 0.04	0.15 ± 0.13	1.08 ± 0.20	1.02 ± 0.20	2.22 ± 0.47	1.23 ± 0.23	0.34 ± 0.05	6.33 ± 1.16
*Flower color effect*			*NS^*c*^*	NS	*NS*	NS	*NS*	NS	NS	*NS*
2017	Annual	Lysidé (w)								
		Silvershadow (w)	0.66 ± 0.06	0.21 ± 0.01	0.43 ± 0.04	0.83 ± 0.07	1.20 ± 0.14	0.88 ± 0.12	0.13 ± 0.02	4.33 ± 0.39
		Fenja (y)	0.27 ± 0.03	0.05 ± 0.01	0.51 ± 0.07	0.76 ± 0.08	1.14 ± 0.14	1.21 ± 0.08	0.11 ± 0.02	4.05 ± 0.41
	Biennial	Jadak (w)	0.30 ± 0.04	0.23 ± 0.03	0.94 ± 0.09	1.05 ± 0.08	1.41 ± 0.13	1.64 ± 0.17	0.15 ± 0.01	5.72 ± 0.52
		Witt (w)	0.26 ± 0.02	0.13 ± 0.06	0.59 ± 0.06	0.83 ± 0.07	1.09 ± 0.10	1.08 ± 0.07	0.06 ± 0.02	4.05 ± 0.25
		Lilput (w)	0.26 ± 0.08	0.13 ± 0.05	0.80 ± 0.25	0.86 ± 0.27	0.95 ± 0.30	1.39 ± 0.44	0.09 ± 0.03	4.48 ± 1.42
		Labrador (y)	0.24 ± 0.01	0.07 ± 0.00	1.06 ± 0.05	1.17 ± 0.08	1.67 ± 0.09	1.75 ± 0.14	0.17 ± 0.01	6.14 ± 0.38
*Flower color effect*			*NS*	^***^	*NS*	*NS*	*NS*	NS	*NS*	*NS*
**SIGNIFICANCE**										
Cv			^***^	^***^	^***^	^**^	^*^	^**^	^***^	^***^
Life cycle			^***^	NS	^**^	^**^	^**^	^*^	^**^	^*^
Year			NS	NS	^**^	NS	^**^	^*^	^***^	NS
Life cycle ^*^ year			NS	NS	^*^	NS	^*^	NS	NS	NS

*Values are means (n = 3) ± standard deviation. Cv significance obtained from f(x) = cv + block; Flower color effect obtained from f(x) = flower color + block within each year; Life cycle and year significance obtained from f(x) = life cycle + year + life cycle × year block*.

a *For abbreviations please consult Table 2. Values of k-3-O-caf-soph-7-O-D-glc and k-3-O-hfer-soph-7-O-D-glc are sums of two isomers*.

b *w, white flower color; y, yellow flower color*.

c NS, not significant;

* p ≤ 0.05;

** p < 0.01;

*** *p < 0.001*.

**Table 9:** Content of hydroxycinnamic acids (mg g^−1^ DM) of seven white or yellow flowering, annual or biennial rapeseed cultivars harvested as baby leaf re-growth (Stage II_cut_) 40 and 60 days after sowing in 2016 and 2017, respectively.

**Year**	**Life cycle**	**Cultivar (cv)**	**Ferulic acid**	**Sinapic acid**	**Caffeoyl- quinic acid**	**Caffeoyl glucoside**	**Disinapoyl-gentiobiose**	**Sinapoyl-feruloyl- gentiobiose**	**Trisinapoyl-gentiobiose**	**Disinapoyl-feruloyl-gentiobiose**	**Total hydroxy-cinnamic acids**
2016	Annual	Lysidé (w)*^*a*^*	0.83 ± 0.24	1.24 ± 0.29	0.63 ± 0.18	0.14 ± 0.05	0.07 ± 0.03	0.01 ± 0.00	0.05 ±	0.03 ± 0.01	3.09 ± 0.83
		Silvershadow (w)									
		Fenja (y)	0.40 ± 0.03	0.73 ± 0.04	0.31 ± 0.00	0.08 ± 0.00	0.14 ± 0.02	0.01 ± 0.00	0.05 ±	0.03 ± 0.00	1.91 ± 0.02
	Biennial	Jadak (w)	0.39 ± 0.03	0.69 ± 0.04	0.35 ± 0.03	0.33 ± 0.02	0.28 ± 0.04	0.02 ± 0.00	0.04 ±	0.03 ± 0.01	2.34 ± 0.08
		Witt (w)	0.69 ± 0.03	0.88 ± 0.04	0.23 ± 0.01	0.25 ± 0.01	0.13 ± 0.01	0.01 ± 0.01	0.04 ±	0.03 ± 0.00	2.36 ± 0.06
		Lilput (w)	0.44 ± 0.03	0.69 ± 0.02	0.34 ± 0.02	0.20 ± 0.01	0.31 ± 0.01	0.00 ± 0.00	0.04 ±	0.03 ± 0.00	2.28 ± 0.04
		Labrador (y)	0.51 ± 0.10	0.80 ± 0.15	0.32 ± 0.07	0.30 ± 0.07	0.38 ± 0.11	0.01 ± 0.02	0.05 ±	0.04 ± 0.01	2.69 ± 0.53
*Flower color effect*			*NS^*b*^*	*NS*	*NS*	*NS*	*NS*	*NS*	^*^	^*^	*NS*
2017	Annual	Lysidé (w)									
		Silvershadow (w)	0.57 ± 0.06	1.20 ± 0.13	0.10 ± 0.01	0.11 ± 0.01	0.03 ± 0.00	0.00 ± 0.00	0.02 ± 0.00	0.01 ± 0.00	2.10 ± 0.22
		Fenja (y)	0.37 ± 0.09	0.91 ± 0.04	0.14 ± 0.02	0.05 ± 0.01	0.11 ± 0.01	0.00 ± 0.00	0.03 ± 0.00	0.01 ± 0.00	1.81 ± 0.16
	Biennial	Jadak (w)	0.18 ± 0.05	0.62 ± 0.15	0.30 ± 0.04	0.19 ± 0.03	0.34 ± 0.04	0.00 ± 0.00	0.03 ± 0.01	0.02 ± 0.00	2.10 ± 0.34
		Witt (w)	0.24 ± 0.04	0.60 ± 0.05	0.15 ± 0.01	0.11 ± 0.00	0.20 ± 0.01	0.00 ± 0.00	0.03 ± 0.00	0.02 ± 0.00	1.64 ± 0.07
		Lilput (w)	0.03 ± 0.00	0.28 ± 0.02	0.22 ± 0.02	0.07 ± 0.00	0.44 ± 0.02	0.02 ± 0.00	0.04 ± 0.01	0.02 ± 0.00	1.69 ± 0.11
		Labrador (y)	0.24 ± 0.01	0.74 ± 0.04	0.23 ± 0.02	0.16 ± 0.01	0.48 ± 0.00	0.00 ± 0.00	0.04 ± 0.00	0.02 ± 0.00	2.46 ± 0.11
*Flower color effect*			*NS*	*NS*	*NS*	*NS*	*NS*	*NS*	*NS*	*NS*	*NS*
**SIGNIFICANCE**											
Cv			*^*c*^*	^***^	^***^	^**^	^***^	NS	^***^	^*^	^***^
Life cycle			^***^	^***^	NS	^***^	^***^	NS	NS	^***^	NS
Year			^***^	NS	^***^	^***^	NS	NS	^***^	^***^	^**^
Life cycle ^*^ year			NS	NS	^***^	^*^	NS	NS	^**^	NS	NS

*Values are means (n = 3) ± standard deviation. Cv significance obtained from f(x) = cv + block; Flower color effect obtained from f(x) = flower color + block within each year; Life cycle and year significance obtained from f(x) = life cycle + year + life cycle × year block*.

a *w, white flower color; y, yellow flower color*.

b NS, not significant;

* p ≤ 0.05;

** p < 0.01;

*** *p < 0.001*.

c *Data could not obtain homogeneity of variance*.

**Table 10:** Content of quercetin glycosides (mg g^−1^ DM) of seven white or yellow flowering, annual or biennial rapeseed cultivars harvested as baby leaf re-growth (Stage II_cut_) 40 and 60 days after sowing in 2016 and 2017, respectively.

**Year**	**Life cycle**	**Cultivar (cv)**	**q-3*-O-*soph-7-*O*-glc*^***a***^***	**q-3-*O*-caf-soph-7-*O*-D-glc**	**q-3-*O*-sin-soph-7-*O*-D-glc**	**Total quercetin glycosides**
2016	Annual	Lysidé (w)*^*b*^*	0.05 ± 0.01	0.08 ± 0.03	0.14 ± 0.07	0.27 ± 0.11
		Silvershadow (w)				
		Fenja (y)	0.05 ± 0.00	0.08 ± 0.00	0.16 ± 0.01	0.28 ± 0.01
	Biennial	Jadak (w)	0.03 ± 0.00	0.03 ± 0.00	0.07 ± 0.01	0.14 ± 0.01
		Witt (w)	0.03 ± 0.00	0.13 ± 0.01	0.16 ± 0.02	0.32 ± 0.02
		Lilput (w)	0.03 ± 0.00	0.04 ± 0.00	0.07 ± 0.00	0.14 ± 0.01
		Labrador (y)	0.04 ± 0.01	0.05 ± 0.01	0.09 ± 0.02	0.19 ± 0.03
*Flower color effect*			*NS^*c*^*	*NS*	*NS*	*NS*
2017	Annual	Lysidé (w)				
		Silvershadow (w)	0.03 ± 0.00	0.07 ± 0.00	0.21 ± 0.03	0.32 ± 0.04
		Fenja (y)	0.03 ± 0.00	0.07 ± 0.01	0.20 ± 0.01	0.29 ± 0.02
	Biennial	Jadak (w)	0.03 ± 0.00	0.05 ± 0.01	0.10 ± 0.02	0.18 ± 0.03
		Witt (w)	0.02 ± 0.00	0.08 ± 0.00	0.15 ± 0.01	0.26 ± 0.01
		Lilput (w)	0.02 ± 0.01	0.05 ± 0.01	0.07 ± 0.02	0.13 ± 0.04
		Labrador (y)	0.03 ± 0.00	0.07 ± 0.01	0.12 ± 0.01	0.23 ± 0.01
*Flower color effect*			*NS*	*NS*	*NS*	*NS*
**SIGNIFICANCE**						
Cv			^*^	*^*d*^*	^***^	^***^
Life cycle			^***^	NS	^***^	^***^
Year			^***^	NS	^*^	NS
Life cycle ^*^ year			NS	NS	NS	NS

*Values are means (n = 3) ± standard deviation. Cv significance obtained from f(x) = cv + block; Flower color effect obtained from f(x) = flower color + block within each year; Life cycle and year significance obtained from f(x) = life cycle + year + life cycle × year block*.

a *For abbreviations please consult Table 4*.

b *w, white flower color; y, yellow flower color*.

c NS: not significant;

* p ≤ 0.05;

** p < 0.01;

*** *p < 0.001*.

d *Data could not obtain homogeneity of variance*.

### Effect of Year and Life Cycle on the Flavonoid Glycoside and Hydroxycinnamic Acid Derivative Content

In general, the levels of flavonoid glycosides and hydroxycinnamic acids were higher in 2016 than 2017 at all growing stages, unless otherwise stated (Tables 2–10).

The life cycle effects on the content of kaempferol glycosides at **Stage I** were limited, however kaempferol-3-*O*-caffeoyl-sophoroside-7-*O*-D-glucoside was found at higher levels in the biennial cultivars (Table 2). In contrast, annual cultivars had higher levels of kaempferol-3-*O*-sinapoyl-sophoroside-7-*O*-diglucoside. An interaction between life cycle and year was found for the kaempferol-3-*O*-coumaroyl-sophoroside-7-*O*-D-glucoside content, meaning that biennial cultivars had higher levels in 2016, whereas annual cultivars had higher levels in 2017. Interestingly, the annual cultivars had a higher content of ferulic and sinapic acids, while the content of caffeoylquinic acid, caffeoyl glucoside, and disinapoylgentiobiose were found to be higher in the biennial cultivars (Table 3). Regarding the content of quercetin glycosides, all identified were measured to be higher in the annual cultivars at Stage I (Table 4).

At **Stage II**_**uncut**_, limited effects were observed on the content of kaempferol, as had been observed at Stage I; biennial cultivars contained higher levels of kaempferol-3-*O*-caffeoyl-sophoroside-7-*O*-D-glucoside, but kaempferol-3-*O*-sophoroside-7-*O*-glucoside levels were higher in the annual cultivars (Table 5). The life cycle effects on hydroxycinnamic acid content at Stage II_uncut_ were similar to Stage I, except that no effects were observed for caffeoylquinic acid, and a higher content of disinapoylferuloylgentiobiose was observed in the biennial cultivars (Table 6). Differences between life cycles were limited to the higher levels of quercetin-3-*O*-sophoroside-7-*O*-glucoside and quercetin-3-*O*-sinapoyl-sophoroside-7-*O*-D-glucoside observed in the annual cultivars at Stage II_uncut_ (Table 7).

A second harvest of the baby leaves (**Stage II**_**cut**_) revealed life cycle effects for all identified kaempferol glycosides, except kaempferol-3-*O*-coumaroyl-sophoroside-7-*O*-D-glucoside (Table 8). A higher content of the non-acylated kaempferol-3-*O*-sophoroside-7-*O*-glucoside and the tetraglycoside kaempferol-3-*O*-sinapoyl-sophoroside-7-*O*-diglucoside were found in the annual cultivars; the opposite trend was observed for kaempferol-3-*O*-sinapoyl-sophoroside-7-*O*-D-glucoside and kaempferol-3-*O*-feruloyl-sophoroside-7-*O*-D-glucoside. An interaction between life cycle and year revealed that the biennial cultivars had a higher content of kaempferol-3-*O*-hydroxyferuloyl-sophoroside-7-*O*-D-glucoside only in 2016, whereas in 2017 the annual cultivars contained lower levels of kaempferol-3-*O*-caffeoyl-sophoroside-7-*O*-D-glucoside. Life cycle effects resulted in higher levels of ferulic and sinapic acids, and lower levels of disinapoylgentiobiose and disinapoylferuloylgentiobiose, in the annual cultivars when compared with the biennial cultivars (Table 9). Furthermore, interactions between the life cycle and year revealed that the caffeoylquinic acid content decreased, in the following order: annuals 2016 > biennials 2016 > biennials 2017 > annuals 2017. A different trend was observed for the caffeoyl glucoside content, in the following order: biennials 2016 > biennials 2017 = annuals 2016 = annuals 2017. The content of the affected quercetin glycosides in the plants harvested at Stage II_cut_ were higher in the annual cultivars than in the biennial cultivars (Table 10). Additionally, a higher quercetin-3-*O*-sinapoyl-sophoroside-7-*O*-D-glucoside content was found in 2017 than 2016.

**Figure 1:**
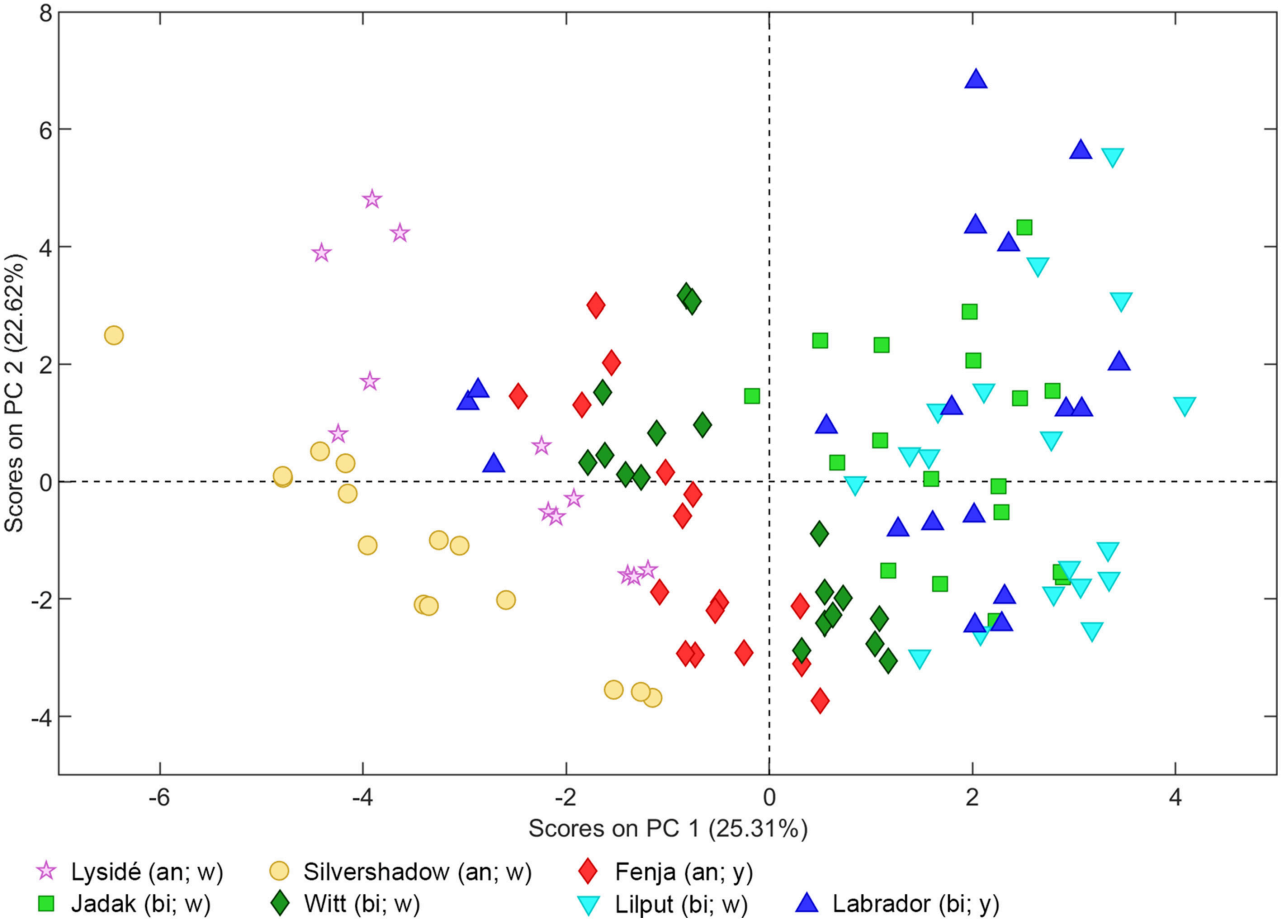
PCA scores plot of flavonoid glycosides and hydroxycinnamic acid derivatives from seven white (w) or yellow (y) flowering cultivars of rapeseed harvested at the growing stages baby leaf (Stage I), baby leaf re-growth (Stage II_cut_), and intact plants (Stage II_uncut_) in 2016 and 2017. An, annual; bi, biennial.

**Figure 2:**
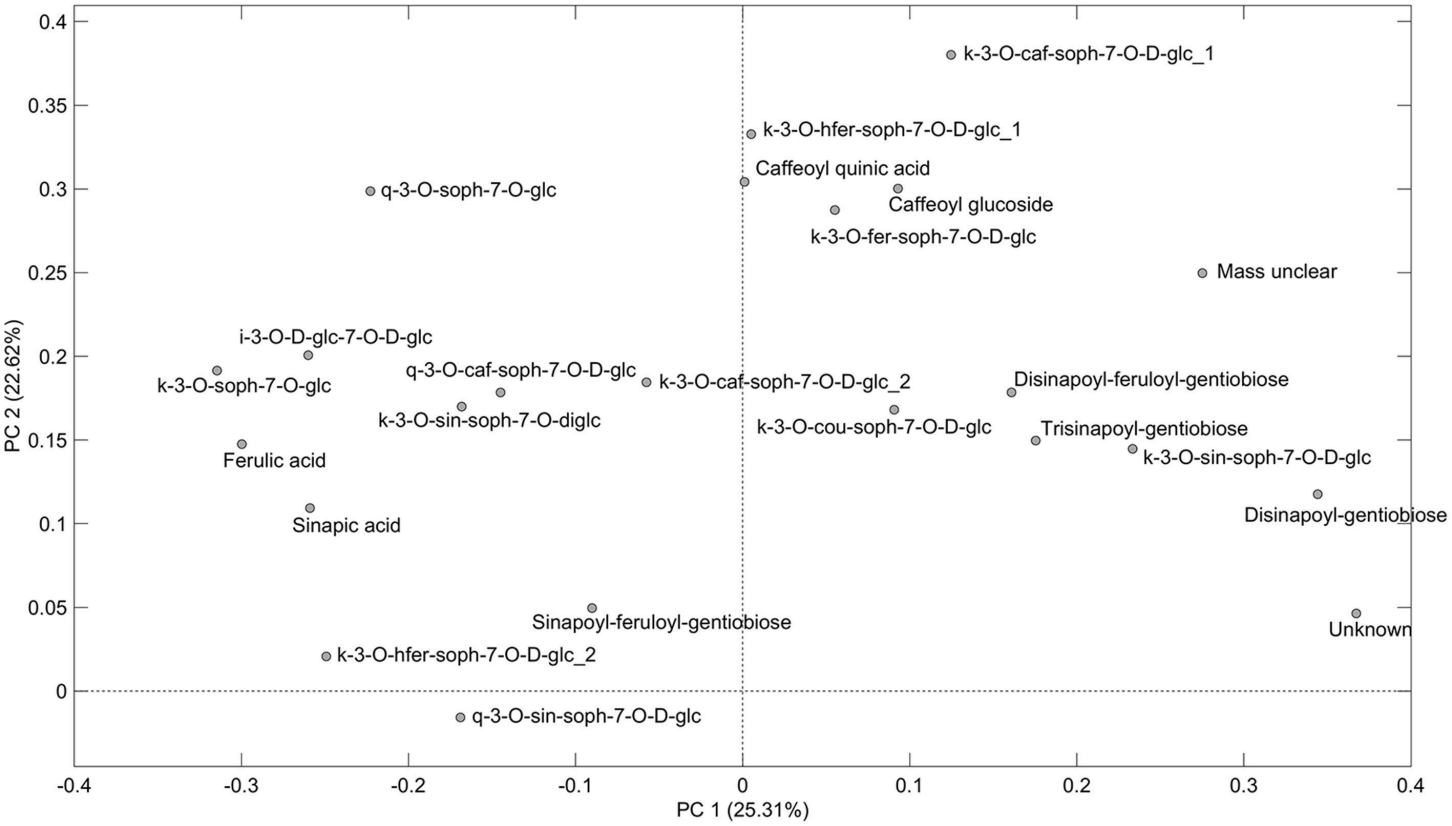
Loading plot of flavonoid glycosides and hydroxycinnamic acid derivatives from seven white or yellow flowering cultivars of rapeseed harvested at the growing stages baby leaf (Stage I), baby leaf re-growth (Stage II_cut_), and intact plants (Stage II_uncut_) in 2016 and 2017. For abbreviations, please consult Table 2.

As can be seen from the PCA scores plot of flavonoid glycosides and hydroxycinnamic acid derivatives from the seven white or yellow flowering cultivars of rapeseed harvested at Stage I, Stage II_cut_, and Stage II_uncut_ in Figure 1, the three annual cultivars “Silvershadow,” “Lysidé,” and partly “Fenja” separate from the biennial cultivars along principal component 1 (PC1). PC1; this means that the life cycle explains 25.3% of the total explained variance in the dataset, followed closely by year (PC2 22.62%). When considering the differences in the glucosylation pattern between annual and biennial cultivars, the biennial cultivars had a higher level of the triglucosylated kaempferol glycosides and the glucosylated hydroxycinnamic acid derivatives than the annual cultivars, as can be seen from the loadings plot (Figure 2). In contrast, the annual cultivars had higher levels of the tetraglucosylated kaempferol glycoside kaempferol-3-*O*-sinapoyl-sophoroside-7-*O*-diglucoside and the quercetin glycosides than the biennials.

### Effect of Growing Stage and Cultivar on the Flavonoid Glycoside and Hydroxycinnamic Acid Derivative Content

**Figure 3:**
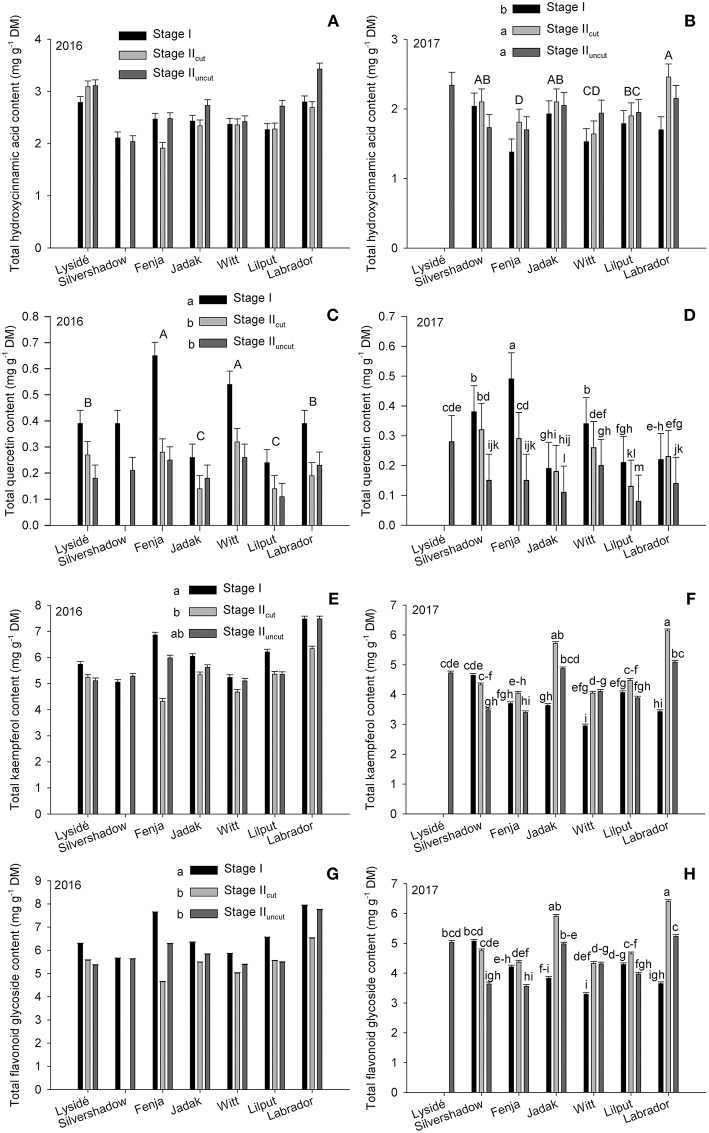
Content of total hydroxycinnamic acid derivatives, quercetin, kaempferol, and total flavonoid glycoside contents (mg g^−1^ DM) in seven white or yellow flowering cultivars of rapeseed at the growing stages baby leaf, Stage I (black bars); baby leaf re-growth, Stage II_cut_ (light gray bars); and intact plants, Stage II_uncut_ (dark gray bars). Note the different scales of y-axes to allow direct comparison of responses. Different letters indicate significant differences (*p* ≤ 0.05) among growing stages (**B,C,E,G**; small letters) and cultivars (**B,C**; capital letters) or cultivar × growing stage **(D,F,H)**. No letters **(A)** indicate no significant differences. When no interaction was found, “Silvershadow” and “Lysidé” were excluded from the statistical calculations due to missing values. Bars represent standard errors (*n* = 3).

The effects of growing stage and cultivar were evaluated within a single year on the total hydroxycinnamic acid derivatives, total quercetin, total kaempferol, and total flavonoid glycosides content (Figure 3). No effect was found for the total hydroxycinnamic acid derivatives content in 2016; however, in 2017, plants harvested at Stage I had a lower content than those harvested at Stage II_cut_ and Stage II_uncut_. Furthermore, an evaluation of cultivar differences revealed that the two yellow flowering cultivars contained the highest (“Labrador,” biennial) and the lowest (“Fenja,” annual) levels of total hydroxycinnamic acid derivatives. In 2016, the effects of growing stages were the same for total quercetin, total kaempferol, and total flavonoid glycosides, which were all presented at higher levels at Stage I. In contrast, interactions were found in 2017 that revealed a higher total quercetin content at Stage I for all cultivars except “Lysidé” and “Labrador,” which was somewhat similar to what was observed in 2016. Moreover, the cultivars responded differently in 2017, with a higher of total kaempferol content observed, primarily in the biennial cultivars “Jadak,” “Witt,” and “Labrador” at Stage II_cut_ and Stage II_uncut_. The annual cultivar “Silvershadow” had a higher total kaempferol content at Stage I and Stage II_cut_, whereas “Fenja” and “Lilput” showed no differences between the growing stages. The same applies to the total flavonoid glycoside content, except that a higher content was also observed at Stage II_cut_ compared to Stage II_uncut_ for “Fenja” and “Silvershadow,” which are both annual cultivars.

## Discussion

### Yields and Growth

The lower number of plants per square meter and the elevated yield in 2016 compared to 2017 could be due to lower competition, and therefore bigger plants (Table 1). Furthermore, these differences reflected the different growing periods and climatic conditions (see Supplementary Figure S1), since the 2016 plants were sown in July, and the 2017 plants were sown in August. A higher average growing temperature (16.9°C in 2016 and 13.7°C in 2017) and higher average radiation (465 μmol m^−2^ s^−1^ in 2016 and 282 in μmol m^−2^ s^−1^ in 2017) resulted in better conditions for photosynthesis and biomass production from sowing till Stage I harvest. Interestingly, although the yield was higher in 2016, and the accumulated GDD before the first harvest was 449 and 388 in 2016 and 2017, respectively, the 2016 plants had not developed further at harvest than the plants from 2017. This can be seen in the similar BBCH-scale codes in Table 1. According to Miller et al. (2001) a range of within 324-463 GDD should lead to a stage with 3-4 unfolded leaves, which corresponds with values of 13 and 14 at the BBCH-scale. The GDD values at the final harvest were 671 (2016) and 741 (2017), which should resemble a flowering stage. However, according to our BBCH-scale registrations made on the intact plants (Stage II_uncut_), only the annual cultivars in 2016 had undergone bud formation, and the buds were visible but closed. One should bear in mind that sowing in July and August is relevant for baby leaf production but not for harvesting seeds later the same year, as would be normal for rapeseed used for oil production in northern Europe. This explains the lack of accordance between the GDD and the suggested developmental stage, as the BBCH-scale was developed for agricultural oil rapeseed.

The harvests at Stage II_cut_ and II_uncut_ were 20 days later in 2017 than in 2016, as it took longer for the harvested plants to develop new leaves due to the lower temperatures and reduced radiation. A day length of 12 h for European spring rapeseed cultivars is required for the initiation of reproductive development in rapeseed (Salisbury and Green, 1991). Therefore, it is possible that no flower buds were seen in the annual cultivars in 2017 since the day length ranged from 11:55 to 9:38 h from the Stage I harvest at the end of September to the final Stage II harvest at the end of October. In comparison, the day length ranged from 16:38 to 13:56 h from sowing to harvest at Stage II in 2016. The biennial cultivars require vernalization in order to move into reproductive development, which did not occur.

In 2016, the annual cultivar “Silvershadow” failed to regrow after the harvest of baby leaves (Stage I), possibly due to the plants being cut below the apical meristem. Furthermore, the biennial plants could be better able to regrow, as they possess the ability to use carbohydrates stored in the root, as has been argued by Hall et al. (2012). In 2017, the annual cultivar “Lysidé” germinated remarkably more slowly, and with a lower germination percentage, than the other cultivars, resulting in no harvest at Stage I for this cultivar. This indicates that the annual cultivars did not perform as well as the biennial under the present conditions. Hall et al. (2012) argued that annual cultivars of rocket (*Eruca sativa*) would be better for cultivation in winter as they develop more rapidly and produce higher yields than the perennials. This was not the case in the present study.

### Effect of Cultivar and Flower Color on the Flavonoid Glycoside and Hydroxycinnamic Acid Derivative Content

Cultivar effects on flavonoid glycoside and hydroxycinnamic acid content were expected, as differences between cultivars are often a major source of variation (Neugart et al., 2018). Variations between cultivars were found at all developmental stages (Tables 2–10). In the present study, the genetic background for the white flowering cultivars were known, as only the annual white flowering cultivars “Lysidé” and “Silvershadow” share the double recessive gene from the cultivar “Hobson” that causes the white petal color. Apart from that, “Lysidé” and “Silvershadow” are not closely related, and are also not closely related to the yellow flowering annual cultivar “Fenja”. In contrast, for the biennial cultivars, at least 62% of the genes of “Witt” and 87.5% of the genes of “Jadak,” and “Lilput” are the same, due to backcrossing with “Labrador” (E. Tybirk, pers. comm.). The divergent degree of kinship among cultivars was comfirmed by the PCA scores plot, where the three annual cultivars, “Silvershadow,” “Lysidé,” and partly “Fenja,” separate from the biennial cultivars along PC1. The flavonoid glycosides and hydroxycinnamic acid derivatives responsible for the observed separation were kaempferol-3-*O*-sophoroside-7-*O*-glucoside, ferulic acid, sinapic acid, and disinapoylgentiobiose (Figure 2), which corresponds to the observed differences in Tables 2, 3, 5, 6, 8, 9. As the biennial cultivars “Jadak” and “Lilput” share 87.5% of their genes with “Labrador,” this resulted in the three cultivars overlapping along PC1. Furthermore, the flavonoid glycosides and hydroxycinnamic acid derivatives found in the biennial cultivar “Witt” show a closer relation to the annual cultivars than to the other biennial cultivars, probably due to its more distant relationship to “Labrador”. Compared with previous studies of different kale cultivars, the variation in flavonoid glycoside content between the rapeseed cultivars in the present study is smaller (Schmidt et al., 2010a; Mageney et al., 2017). As the modern cultivars of rapeseed that possess the double low qualities (low contents of glucosinolates and erucic acid in the seed) were introduced during the 1970's, the modern rapeseed cultivars represent a relatively young crop compared to the traditional *B. oleracea* cabbages (Stefansson, 1983). Therefore, the current rapeseed cultivars may be less differentiated and distinct, as selection and breeding has not been carried out for as long as for *B. oleracea* cabbages and kales.

The effect of flower color on the content of flavonoid glycosides and hydroxycinnamic acid derivatives in the leaves were inconsistent, and thus suggested that no direct connection can be concluded. However, our analyses focused on the leaves and not the petals, and focusing on the petals might provide a different result. Previous investigations of the relationship between alleles that determine the petal color of soybean (*Glycine max* L. Merr.) and the flavonoid content revealed a connection (Iwashina et al., 2008). Thus, a lower content of the anthocyanins malvidin-3.5-di-*O*-glucoside, petunidin-3.5-di-*O*-glucoside, delphinidin-3.5-*O*-glucoside, and delphinidin-3-*O*-glucoside was found in the soybean line with near-white flowers.

This indicates that anthocyanins are absent in rapeseed petals, and that the flavonoid content might affect the rapeseed petal color. However, Zhang et al. (2015) reported that rapeseed petal color is regulated by carotenoid biosynthesis, and previous analyses of the rapeseed cultivars used in this study showed a higher content of total carotenoids in the yellow flowering cultivars when grown as seedlings (Groenbaek et al., 2018).

### Effect of Year and Life Cycle on the Flavonoid Glycoside and Hydroxycinnamic Acid Derivative Content

When an effect due to year was found, the levels of the respective hydroxycinnamic acid derivatives or the flavonoid glycoside groups were always higher in 2016 than in 2017, with the exception of two cases (Tables 2–10). This difference was perhaps due to the climate differences between the 2 years, as the sowing in 2016 took place 5 weeks earlier (July 20th) than in 2017 (August 28th). Therefore, the daily average temperature and radiation was higher for the plants grown in 2016 (Supplementary Figure S1). The effects of radiation on hydroxycinnamic acid derivatives and flavonoid glycosides has previously been investigated in kale, and it has been shown that higher levels of quercetin flavonoid glycosides, and lower levels of kaempferol flavonoid glycosides, were caused by the higher levels of radiation (Neugart et al., 2016). In another study on kale, the opposite effect was seen, with lower quercetin levels and higher kaempferol levels at higher radiation levels (Schmidt et al., 2010a). The previously mentioned experiment was conducted during the autumn and winter as a field experiment with decreasing temperatures occurring concurrently with the decreasing radiation, arguing that temperature in this case suppressed the effect of radiation (Schmidt et al., 2010a). In contrast, the elevated quercetin levels were found at a constant temperature of 10°C in a climate chamber with higher levels of radiation, as mentioned above (Neugart et al., 2016). In the present study, mean temperatures were higher in 2016 than 2017, and were as much as double the temperatures observed in the previously mentioned studies (Supplementary Figure S1). It seems likely that temperatures in the present study were affecting flavonoid biosynthesis to a lesser extent. In a study on lettuce (*Lactuca sativa*) grown under average temperatures, ranging from 15.6 to 21.2°C, the total flavonoids and phenolics content were lower due to lower radiation since the experiments were conducted with shifted sowing and transplantation (June and July), similar to our study (Tsormpatsidis et al., 2008).

We found a higher level of the triglucosylated kaempferol glycosides and the glucosylated hydroxycinnamic acid derivatives in the biennial cultivars, whereas the annual cultivars had higher levels of the tetraglucosylated kaempferol-3-*O*-sinapoyl-sophoroside-7-*O*-D-glucoside and the quercetin glycosides (Figure 2). In comparison, a study on the annual salad rocket (*Eruca sativa* L.) and the biennial wall and wild rockets (*Diplotaxis erucoides* L. and *D. tenuifolia* L.) exhibited the opposite pattern, with higher levels of di- and tri-glucosylated kaempferol glycosides detected in the annual cultivars, and only tri- and tetra-glucosylated quercetin glycosides detected in the biennials (Bennett et al., 2006). Biennial cultivars are designated to overwinter in the field, and to not start flowering until the following spring. Therefore, an increase in reactive oxygen species (ROS) due to lower temperatures and plant aging could lead to a greater requirement for phytochemicals with a direct antioxidant capacity, such as quercetin glycosides (Vogt and Gerhard Gul, 1994; Klimov et al., 2008). This effect was observed in kale grown in the field in northern Germany from July till January (Zietz et al., 2010). In particular, this was confirmed by the higher content of disinapoylgentiobiose and kaempferol-3-*O*-caffeoyl-sophoroside-7-*O*-D-glucoside in the biennial cultivars harvested at Stage II_uncut_, as previous findings revealed an antioxidant capacity of 4.5 and 1.15 (Zietz et al., 2010), respectively, for the above-mentioned compounds. In comparison, the quercetin flavonoid glycosides at the same stage had an antioxidant activity of 1.9 (quercetin-3-*O*-sophoroside-7-*O*-glucoside) and 2.29 (quercetin-3-*O*-sinapoyl-sophoroside-7-*O*-D-glucoside) (Zietz et al., 2010).

### Effect of Growing Stage and Cultivar on the Flavonoid Glycoside and Hydroxycinnamic Acid Derivative Content

It has previously been suggested that ontogeny has an effect on the content of the respective aglycones, and we found effects due to the growing stage, but this was also dependent on the year (Figure 3). In kale grown in the field late summer/early autumn in Denmark, kaempferol and total flavonoid glycosides increased in plants from 8 to 13 weeks of age, whereas no effects were found for quercetin (Groenbaek et al., 2016). In pak choi (*B. rapa* subsp. *chinensis*), the levels of quercetin glycosides were higher in plants with five leaves than in plants with 15 leaves, whereas the levels of kaempferol glycosides were in general higher in plants with 30 leaves, depending on the acylation pattern (Heinze et al., 2018). The five and 15 leaves growing stages were somewhat comparable to stages I and II_uncut_ in the present study, and supports our results since the levels of quercetin glycosides were lower at Stage II_uncut_ than at Stage I (Figures 3C,D). In more detail, an estimation of the content of total quercetin glycosides found by Heinze et al. (2018) showed that the 5-leaf pak choi plants contained approx. 1.8 mg g^−1^ DM and the 15-leafplants contained approx. 0.6 mg g^−1^ DM. These values were twice the content of the rapeseed cultivars with the highest content (Tables 4, 7), which is possibly due to the identification of more individual quercetin glycosides in the pak choi compared with the rapeseed. The content of total kaempferol in the 5-leaf plants were estimated to be approx. 4.7 mg g^−1^ DM and 3.64 mg g^−1^ DM in the 15-leaf plants. Levels that were in the mid-range and lower end compared to the tested rapeseed cultivars in the present study harvested at the comparable stages, as mentioned above (Tables 2, 5). The only hydroxycinnamic acid identified in both studies was caffeoyl-quinic acid whose levels were estimated to be 0.12 and 0.19 mg g^−1^ DM in the 5-leaf and 15-leaf plants, respectively. Compared to the levels in the different rapeseed cultivars, these were the same as the cultivars with the lowest levels (Tables 3, 6).

The effect of growing stage on quercetin glycosides in the present study was comparable between years, in contrast to what was observed for kaempferol (Figures 3C–F). This could indicate that ontogenetic effects were more critical for determining quercetin biosynthesis in rapeseed than the influence of the climate, compared to kaempferol. A similar result was found by Becker et al. (2014) where decreasing quercetin levels was seen with plant ontogeny in lettuce (*Lactuca sativa* L. var. *crispa*) but less sensitivity to radiation intensity and thereby climate, was found. Furthermore, several interactions between developmental stage and UV radiation treatment that influenced the kaempferol glycosides content in pak choi were found, whereas only effects due to developmental stage were observed for the quercetin glycosides (Heinze et al., 2018).

Flavonoid glycosides have previously been suggested to be able to reduce ice formation in plant cells, due to the osmoregulatory effect of the glycosides, which is similar to that of soluble sugars. Furthermore, the large number of hydroxyl groups could all form bonds with water (Swiderski et al., 2004). The ability to lower the osmotic potential is well known from winter hardy plants and is necessary to survive potential frost and the subsequent damage that the biennial cultivars experience during winter. This is supported by the higher levels of kaempferol glycosides and disinapoylgentiobiose observed in the biennial cultivars harvested at Stage II in 2017 (Figures 3B,F). An increase in flavonoids in cold-acclimatized *Arabidopsis* was found when compared to non-acclimatized plants (Korn et al., 2008). However, a previous study found an unchanged level of flavonoid glycosides in field-grown kale experiencing similar conditions as the rapeseed in the present study experienced in 2017 (Groenbaek et al., 2016). In that study, the flavonoid glycoside content increased in plants at age 8 to 13 weeks, followed by a decrease to the same level as plants at 8 weeks age in plants at 17 weeks age.

Investigations in to the effect of repeated harvest on flavonoid glycosides and hydroxycinnamic acid derivatives are scarce (Letchamo, 1996; Guldbrandsen et al., 2015; Sasaki et al., 2015). A study on the effect of repeated harvests on caffeoylquinic acid in edible sweet potato leaves (*Ipomoea batatas* (L.) Lam.) revealed a decrease in mono-caffeoylquinic acids in the second, third, and fourth harvest, compared to the first (Sasaki et al., 2015). In contrast, the second harvest of chamomile (*Chamomilla recutita* (L.) Rauschert) flowers had a higher content of the flavonols apigenin and apigetrin compared to the first, third, and fourth harvests (Letchamo, 1996). In the present study, the Stage I harvest, from which the re-growth took place, was a reduction in leaf material of at least 75%. It also seems likely that the root system would be heavily reduced in size (Kristensen and Stavridou, 2017). In general, defoliation reduces the CO_2_ assimilation of the plant, and the initial N storage in the rosette, leaving the plant with little photosynthetic tissue or carbohydrates accumulated in the roots. Reduced N application to kale and low N concentrations in the leaves of *B. rapa* and *B. juncea* have been shown to increase levels of quercetin, kaempferol, and total flavonoid glycosides (Fallovo et al., 2011; Groenbaek et al., 2014). However, as different responses to repeated harvest were observed between the 2 years, seasonal effects likely determined the biosynthesis of the kaempferol glycosides and hydroxycinnamic acids to some degree. The later growing season in 2017 might have triggered a greater requirement for the antioxidant capacity due to the lower temperatures and the longer growing period, as discussed above. Furthermore, the capability to utilize the stored carbohydrates in the roots of the biennial cultivars (Hall et al., 2015) and the fact that the biennial cultivars were under normal growing conditions compared to the annual cultivars in 2017, possibly lead to there being enough resources for both primary and secondary metabolism in the biennial cultivars (Figures 3B,F,H).

In conclusion, white and yellow flowering cultivars of rapeseed proved suitable for cultivation of baby leaves with repeated harvest. Repeated harvest is recommended for a more sustainable production with no consistent impact on the phytochemical contents, as shown here. With the aim of increasing the content of flavonoid glycosides and hydroxycinnamic acids, the choice of life cycle and season effects was of major influence, overruling the effect of flower color and developmental stage, except for the quercetin content within the developmental stage.

## Data Availability

All datasets generated for this study are included in the manuscript and/or the Supplementary Files.

## Author Contributions

MG secured the financing, designed the experiments, collected data, analyzed the results and drafted the manuscript. ET and HK contributed with practical and research experience and revising of the manuscript. SN managed the chemical analysis and US did the PCA statistics. MS reviewed the final manuscript and secured the chemical analysis. All authors have read and approved the final manuscript.

### Conflict of Interest Statement

Knold and Top ApS. has contributed to the research grant given to author MG, in addition to the donation of seeds and knowledge. The remaining authors declare that the research was conducted in the absence of any commercial or financial relationships that could be construed as a potential conflict of interest.

## Supplementary Material

Supplementary Figure S1 Climatic data from the two growing seasons, 2016 and 2017. Daily average temperature (°C), daily precipitation (mm), and daily average radiation (μmol m^−2^ s^−1^). Arrows indicate baby leaf harvest (Stage I), 27 (2016) and 29 (2017) days after sowing (DAS). End of data points indicate harvest at Stage II (baby leaf re-growth and intact plants) 40 and 60 DAS in 2016 and 2017, respectively.

## Abbreviations

DAD: diode array detector
DAS: days after sowing
DM: dry matter
ESI: electrospray ionization
GDD: growing degree days
HPLC: high-performance liquid chromatography
MS^n^: mass spectrometry
PC: principal component
PCA: principal component analysis
UV: ultraviolet.
