# Supplementary figures and images for: Flavonoid Glycosides and Hydroxycinnamic Acid Derivatives in Baby Leaf Rapeseed From White and Yellow Flowering Cultivars With Repeated Harvest in a 2-Years Field Study
Source: Front Plant Sci. 2019 Apr 2;10:355. doi: 10.3389/fpls.2019.00355 (PMC6454053; doi:10.3389/fpls.2019.00355)

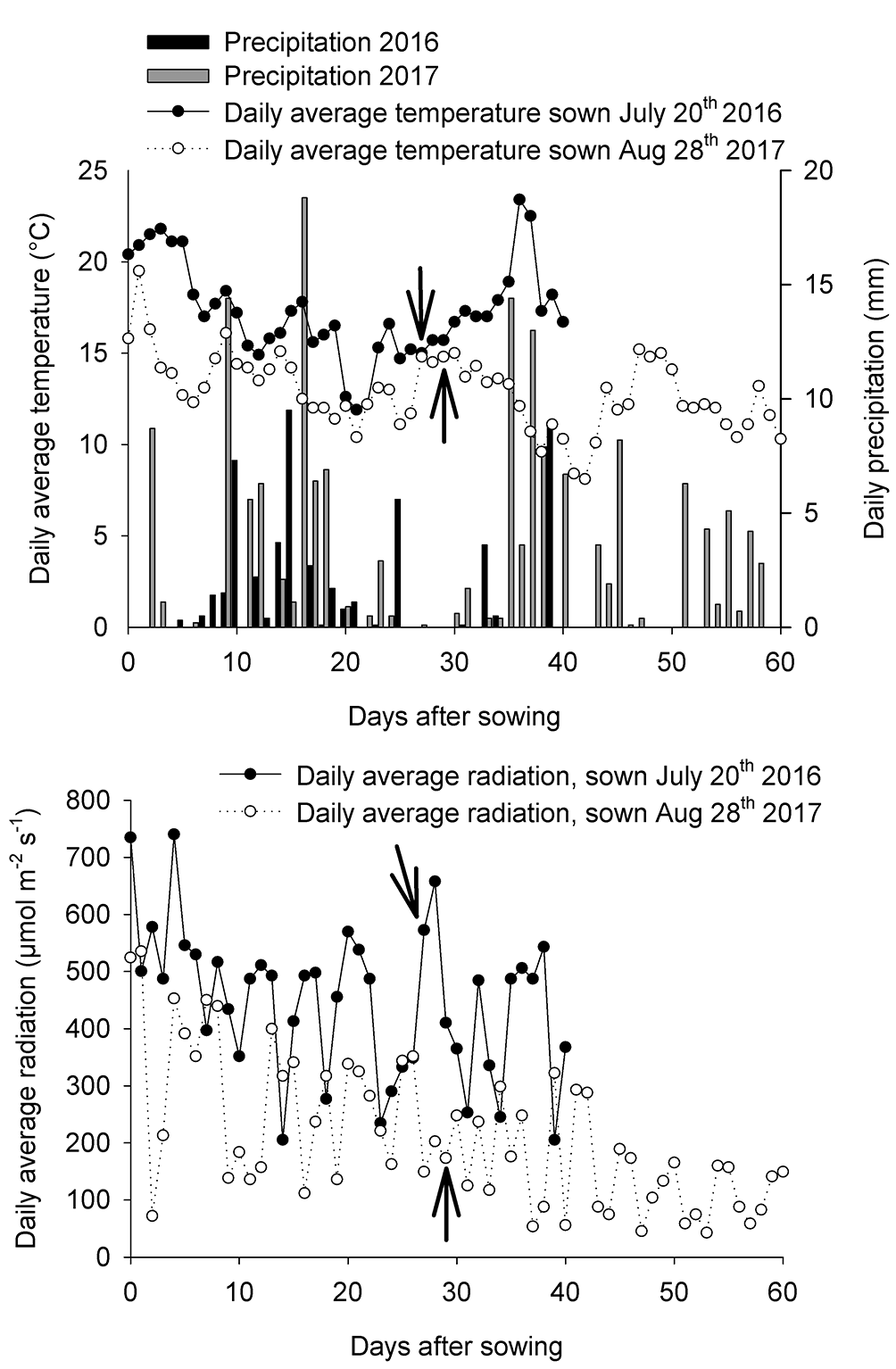

Supplement: Supplementary Figure S1 — Climatic data from the two growing seasons, 2016 and 2017. Daily average temperature (°C), daily precipitation (mm), and daily average radiation (μmol m−2 s−1). Arrows indicate baby leaf harvest (Stage I), 27 (2016) and 29 (2017) days after sowing (DAS). End of data points indicate harvest at Stage II (baby leaf re-growth and intact plants) 40 and 60 DAS in 2016 and 2017, respectively. [file Image_1.TIF]
